# Knowledge and Awareness of Rare Diseases Among Healthcare Professionals in the Kingdom of Bahrain

**DOI:** 10.7759/cureus.47676

**Published:** 2023-10-25

**Authors:** Israa Sinan, Maha Mihdawi, A. Rahman Farahat, Mariam Fida

**Affiliations:** 1 Research and Development, King Hamad University Hospital, Al Muharraq, BHR; 2 Research and Development, Canadore College, North Bay, CAN; 3 Nursing Research, King Hamad University Hospital, Al Muharraq, BHR; 4 Pathology and Laboratory Medicine, King Hamad University Hospital, Al Muharraq, BHR; 5 Medical Genetics, Bahrain Oncology Centre, King Hamad University Hospital, Al Muharraq, BHR

**Keywords:** medical education, rare diseases, genetic literacy, health literacy, genomics, genetics, continuing education

## Abstract

Aim

Recent studies highlighted that lack of knowledge on rare diseases is a problem that requires attention. This study aims to assess healthcare professionals' general awareness and knowledge of rare diseases in a tertiary hospital in the Kingdom of Bahrain.

Method

The study employed a cross-sectional design, utilizing a survey questionnaire derived from the most recent literature. The survey encompassed socio-demographic factors and quiz-based questions that were previously created by Domaradzi and Walkowiak to assess knowledge and awareness of rare diseases. To ensure convenience and accessibility, the survey was made available in both Arabic and English languages.

Results

Of a total of 333 responses, 25.2% were physicians, 53.8% were nurses, and 21.0% were allied health personnel. The majority of participants (87.4%) were aware of and had heard the term "rare diseases" prior to this survey. Participants were able to recognize what age group is frequently affected by rare diseases (p=0.023) and what the common cause of rare diseases worldwide is (p<0.001). Overall scores showed that only four participants answered all questions correctly, testing their knowledge of rare diseases. There was a weak correlation between self-declared knowledge and the overall score achieved (r=0.190; p<0.001), which indicates that the population's self-declared knowledge did not portray their actual knowledge of rare diseases.

Conclusion

This study highlights the need for improved knowledge of rare diseases among healthcare professionals, which aligns with the global knowledge landscape. To bridge the knowledge gap, we recommend action plans to ensure that healthcare professionals have rich knowledge of rare diseases and further improve patient care. Additionally, enhancing advocacy efforts is crucial to ensure optimal local and global patient care services.

## Introduction

Rare diseases could be defined as a set of disorders that only affect a small number of people [1]; however, they tend to be prevalent in every clinical setting. There are more than 7,000 classified rare diseases [2], and the exact definition of rare diseases differs between regions. The Saudi Ministry of Health has stated that a disease is defined as rare when it affects less than 1 per 2000 people [2]. The Kingdom of Bahrain has no set definition for rare diseases. However, the advocacy for such diseases has seen increased awareness campaigns such as Rare Disease Day and the National Genome Project by the Ministry of Health. Enormous challenges were noted in the global rare diseases research communities, such as prompt diagnosis, approved treatments available and access to them, and improved quality of life for rare disease patients. In 2011, the International Rare Diseases Research Consortium (IRDiRC) was founded to aid in developing new therapies and the means to diagnose most rare diseases [3]. In other words, the IRDiRC has played a significant role in raising public awareness about rare diseases and the need to address them. Healthcare professionals will face diagnosing or treating a rare disease at some point in their professional career. Recent studies highlighted that lack of knowledge on rare diseases is a problem that requires attention. In China, only 5.3% of physicians interviewed were moderately or well aware of rare diseases [4]. In Spain, only 15% of physicians were moderately or well aware of rare diseases [5]. Similar studies were conducted on medical students, in particular, a study done in Egypt on pharmacy and medical students in 2020. This study found that 97% of students wanted a broader look into rare diseases [6]. Similarly, other studies mentioned, it was found that only 11.76% of medical students rated their knowledge as very good about rare diseases [6]. Therefore, this indicates that rare diseases are often overlooked by medical education, thus leading to delayed diagnosis, misdiagnosis, or improper treatment, which results in poor patient care. This study aims to develop a baseline on the healthcare workers' knowledge and awareness of rare diseases. In addition, this will help identify any gap that exists to provide those with rare diseases with better patient-centered care at King Hamad University Hospital and Bahrain Oncology Center.

## Materials and methods

Design, sample, sampling, and setting

The study employed a cross-sectional design, utilizing a survey questionnaire derived from the most recent literature. The survey encompassed socio-demographic factors and quiz-based questions that Walkowiak D and Domaradzki J previously created to assess knowledge and awareness of rare diseases. To ensure convenience and accessibility, the survey was made available in both Arabic and English languages. This study aimed to include physicians, nurses, and allied health personnel with a minimum of three months' work experience at a tertiary educational hospital in Bahrain, excluding administration staff. Educational background for physicians that were included in the study had a minimum of a Medical Degree, and nurses had a minimum of a Bachelor's in Nursing. The Allied Health personnel's educational background varied based on the profession, including a minimum of Bachelor's in Pharmacy, Laboratory Science, Physiotherapy, or Radiology Science. To obtain a 95% CI with 5% marginal error, a total of 323 responses was the estimated sample size required for this study. 

Measures 

The primary objective of this study is to assess knowledge and awareness of rare diseases among healthcare professionals. The secondary objective of this study is to compare the knowledge among different occupations. Most of the studies used a quiz-based survey that was developed by Walkowiak D and Domaradzki J from Poland [7-9]. The survey was reported to be reliable, using the internal consistency measure with Cronbach's alpha where α = 0.76 [8]. The current study reports a Cronbach's alpha of 0.71. This quiz-based survey compromised two components: the socio-demographic characteristics of the respondents and the self-assessment of knowledge about rare diseases. The socio-demographical questionnaires vary based on countries. For example, in the study conducted in Kazakhstan, they segregated years of experience differently than in the original study in Poland [10]. In the study conducted in Egypt, they inquired about the marital status of participants [6]. This quiz-based survey was adopted and modified to be used in the study. The two sections were: 

Socio-demographical data such as age, gender, marital status, years of experience, occupation (Physician, Nurse, and Allied Health), educational background (local, abroad), if they have met a person suffering from a rare disease, and if anyone is suffering from a rare disease in their family. Knowledge and perception about rare diseases: The survey was created based on previous literature and combined questions from physicians, nurses, and students' assessments [7-9]. The survey was modified to include only general questions on the knowledge and awareness about rare diseases (excluding any country-specific questions). There were no added questions to the original material. The survey was translated to Arabic for ease of understandability and accessibility by staff at all levels and was available in both languages. The reliable double translation method was followed to guarantee attaining semantic equivalence between the two languages: firstly, forward translation from English to Arabic; secondly, an author independently performed the back-translation into English. Then, a third bilingual expert compared the original quiz and the back-translated one for consistency in meaning and adjusting as required. The survey was then formatted into Microsoft Forms and circulated to the hospital weekly for a duration of five weeks. 

Ethical consideration and data collection

This study was reviewed and approved by the Institutional Review Board at King Hamad University Hospital (#*23-580*).

Before participating, the study was explained to all participants through an informed consent section at the beginning of the questionnaire. This section provided a brief description of the study, the names and contact details of the investigators, a note on voluntary participation, and a statement about anonymity and the right to withdraw. Participants could only access the rest of the questionnaire after clicking the consent button, which acted as a signature. Those who did not give their consent were automatically directed to the survey's end. The questionnaire took approximately five minutes to complete.

Data analysis plan 

Data were analyzed using SPSS version 25 (IBM Corp., Armonk, NY, USA). Basic descriptive statistics, including frequencies and percentages for categorized data and means and SD for continuous data, were used to describe participants' socio-demographics and their responses. For each knowledge question, the correct answer was coded as one, while all incorrect answers were coded as zero. The Chi-square test was used to assess differences in the distribution of answers among groups. Spearman's correlation test was used to examine the correlation between self-declared knowledge and the total achieved score. A p-value of less than 0.05 was considered significant.

## Results

There was a total of 350 responses, of which only 333 consented to complete the survey over a duration of five weeks. The responses were divided into three categories that defined the participants' occupations. The first category was physicians, the second category was nurses, and the third category was allied health personnel. There was a total of 84 (25.2%) physicians, 179 (53.8%) nurses, and 70 (21.0%) allied health personnel that completed the survey (Table 1). 

**Table 1:** Participants’ demographics.

Variable		Physicians (n=84)	Nurses (n=179)	Allied Health (n=70)	Total (n=333)
		Mean ± SD (Min-Max)			
Age (years)		35.29±11.0 (22-67)	33.35±6.7(22-60)	32.7±8.3 (21-61)	33.70±8.3 (21-67)
Work Experience (years)		9.56±10.2 (0.58-43)	8.73±6.2(0.25-31)	9.19±6.8 (0.25-38)	9.05±7.5 (0.25-43)
		n (%)			
Gender	Male	43 (51.2%)	40 (22.3%)	12 (17.1%)	95 (28.5%)
	Female	41 (48.8%)	139 (77.7%)	58 (82.9%)	238 (71.5%)
Degree Obtained	Local	41 (48.8%)	45 (25.1%)	42 (60.0%)	128 (38.4%)
	Abroad	43 (51.2%)	134 (74.8%)	28 (40.0%)	205 (61.6%)
Marital Status	Single	36 (42.9%)	37 (20.7%)	30 (42.9%)	103 (30.9%)
	Married	45 (53.6%)	133 (74.3%)	40 (57.1%)	218 (65.5%)
	Divorced	2 (2.4%)	5 (2.8%)	0	7 (2.1%)
	Widow	0	2 (1.1%)	0	2 (0.6%)
	Separated	1 (1.1%)	2 (1.1%)	0	3 (0.9%)
Have you met someone suffering from a rare disease?	Yes	57 (67.9%)	68 (38.0%)	29 (41.4%)	154 (46.2%)
	No	18 (21.4%)	88 (49.2%)	29 (41.4%)	135 (40.5%)
	I don’t know	9 (10.7%)	23 (12.8%)	44 (62.9%)	44 (13.2%)
Is anyone from your family suffering from a rare disease?	Yes	8 (9.5%)	12 (6.7%)	7 (10.0%)	27 (8.1%)
	No	73 (86.9%)	160 (89.4%)	60 (85.7%)	293 (88.0%)
	I don’t know	3 (3.6%)	7 (3.9%)	3 (4.3%)	13(3.9%)

**Figure 1:**
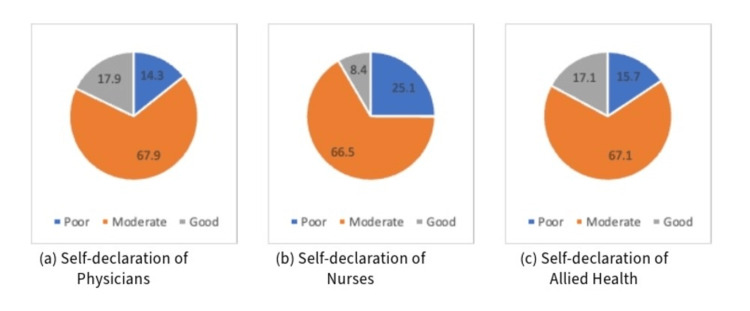
Self-declaration of knowledge by participants: (a) physicians, (b) nurses, and (c) allied health professionals.

Demographics were not strong indicators of the population's knowledge about rare diseases, as analysis did not show any significance. In specific, there was no significance (p=0.287) between where the participants obtained their degree and their awareness of rare diseases. Participants were asked to self-declare their knowledge of rare diseases categorically by indicating if they had poor, moderate, or good knowledge (Figure 1). Self-declaration showed significance between occupations (p=0.044), and this was also noted between occupations when participants were asked about their previous knowledge attained through their educational background (p=0.006). Most participants (92.8%) also indicated that they would like to broaden their knowledge of rare diseases. 

As part of the initial survey [7-9], the first question collected participant's awareness of rare diseases. Many participants (87.4%) had heard the term "rare diseases" previously. This was also proven to be significant (p<0.001) in this population. Seven questions were taken from the original survey to assess the knowledge of rare diseases (Table 2). 

**Table 2:** Participants' answers on their knowledge of rare diseases. Note: correct answers are indicated in bold. * indicates significant value.

Questions and answers	Physicians (n=84)	Nurses (n=179)	Allied Health (n=70)	Total (n=333)	P-value
1. A rare disease is one that affects less than:					
1 person in 1000	5 (6.0%)	51 (28.5%)	15 (21.4%)	71 (21.3%)	0.506
1 person in 2000	10 (12.0%)	26 (14.5%)	13 (18.6%)	49 (14.7%)	
1 person in 3000	0	1 (0.6%)	2 (2.9%)	3 (0.9%)	
1 person in 5000	6 (7.1%)	0	4 (5.7%)	10 (3.0%)	
1 person in 10,000	44 (52.4%)	56 (31.3%)	19 (27.1%)	119 (35.7%)	
I don’t know	19 (22.6%)	45 (25.1%)	17 (24.3%)	81 (24.3%)	
2. What is the estimated number of rare diseases worldwide?					
100-500	13 (15.5%)	30 (1.8%)	9 (12.9%)	52 (15.6%)	0.783
1000-2000	7 (8.3%)	11 (6.1%)	6 (8.6%)	24 (7.2%)	
3000-5000	4 (4.8%)	10 (5. 6%)	7 (10.0%)	21 (6.3%)	
6000-8000	9 (10.7%)	21 (11.7%)	10 (14.3%)	40 (12.0%)	
9000-10,000	3 (3.6%)	5 (2.8%)	4 (5.7%)	12 (3.6%)	
Over 10,000	6 (7.1%)	10 (5.6%)	2 (2.9%)	18 (5.4%)	
I don’t know	42 (50%)	92 (51.4%)	32 (45.7%)	166 (49.8%)	
3. At what age group do rare diseases most frequently appear?					
Newborns	19 (22.6%)	22 (12.3%)	14 (20.0%)	55 (16.5%)	0.023*
Children	23 (27.4%)	42 (23.5%)	7 (10.0%)	72 (21.6%)	
Adolescents	2 (2.4%)	4 (2.2%)	2 (2.9%)	8 (2.4%)	
Adults	6 (7.1%)	9 (5.0%)	3 (4.3%)	18 (5.4%)	
They present in all ages equally.	13 (15.5%)	60 (33.5%)	33 (47.1%)	106 (31.8%)	
I don’t know	21 (25%)	42 (23.5%)	11 (15.7%)	74 (22.2%)	
4. How many people suffer from rare diseases worldwide?					
10-50 million	17 (20.2%)	29 (16.2%)	14 (20.0%)	60 (18.0%)	0.545
50-75 million	5 (6.0%)	17 (9.5%)	1 (1.4%)	23 (6.9%)	
100-150 million	6 (7.1%)	9 (5.0%)	4 (5.7%)	19 (5.7%)	
200-250 million	2 (2.4%)	4 (2.2%)	3 (4.3%)	9 (2.7%)	
300-350 million	5 (6.0%)	18 (10.1%)	6 (8.6%)	29 (8.7%)	
Over 500 million	0	3 (1.7%)	1 (1.4%)	4 (1.2%)	
I don’t know	49 (58.3%)	99 (55.3%)	41 (58.6%)	189 (26.7%)	
5. What is the most common cause of rare diseases worldwide?					
Infectious	2 (2.4%)	11 (6.1%)	2 (2.9%)	15 (4.5%)	<0.001
Genetic	62 (73.8%)	77 (43.0%)	39 (55.7%)	178 (53.5%)	
Autoimmune	2 (2.4%)	44 (24.6%)	14 (20.0%)	60 (18.0%)	
Mitochondrial	4 (4.8%)	1 (0.6%)	1 (1.4%)	6 (1.8%)	
Environmental	2 (2.4%)	5 (2.8%)	2 (2.9%)	9 (2.7%)	
I don’t know	12 (14.3%)	41 (22.9%)	12 (17.1%)	65 (19.5%)	
6. What percentage of rare diseases are of genetic origin?					
5-10%	11 (13.1%)	28 (15.6%)	9 (12.9%)	48 (14.4%)	0.653
20%	12 (14.2%)	23 (12.8%)	9 (12.9%)	44 (13.2%)	
50%	9 (10.7%)	21 (11.7%)	9 (12.9%)	39 (11.7%)	
80%	17 (20.2%)	28 (15.6%)	12 (17.1%)	57 (17.1%)	
100%	2 (2.4%)	0	1 (1.4%)	3 (0.9%)	
I don’t know	33 (39.3%)	79 (44.1%)	30 (42.9%)	142 (42.6%)	
7. What percentage of rare diseases can be treated with drugs?					
0%	1 (1.2%)	5 (2.8%)	1 (1.4%)	7 (2.1%)	0.833
5%	17 (20.2%)	40 (22.3%)	17 (24.3%)	74 (22.2%)	
10%	15 (17.9%)	21 (11.7%)	4 (5.7%)	40 (12.0%)	
15%	4 (4.8%)	8 (4.5%)	5 (7.1%)	17 (5.1%)	
20%	6 (7.1%)	11 (6.1%)	4 (5.7%)	21 (6.3%)	
50%	1 (1.2%)	13 (7.3%)	6 (8.6%)	20 (6.0%)	
I don’t know	40 (47.6%)	81 (45.3%)	33 (47.1%)	154 (46.2%)	

**Figure 2:**
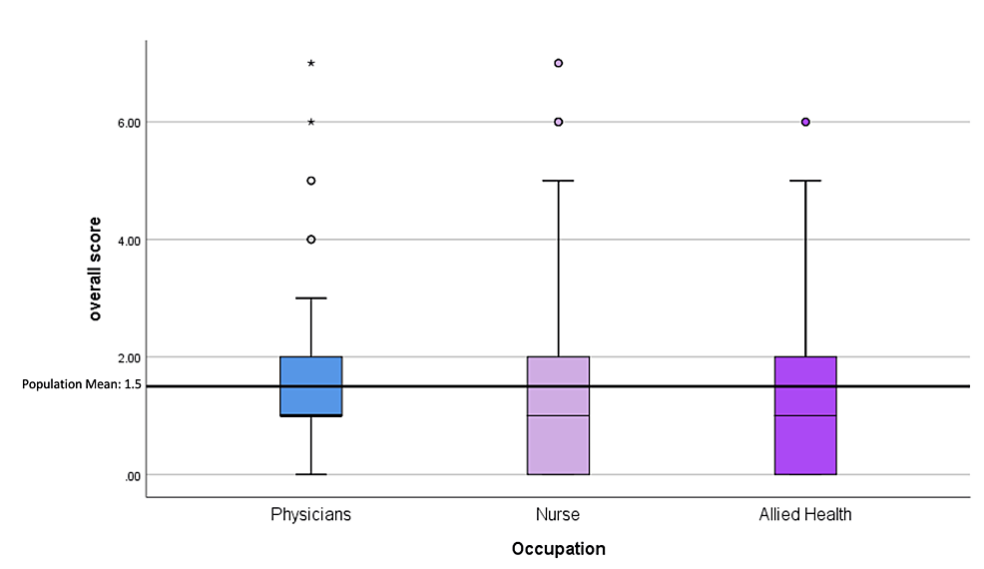
Box and whisker plot of the overall score by occupation.

The significance of the correct answer amongst the wrong answers was analyzed. As seen above, participants across all three occupations showed that their knowledge of what age group rare diseases frequently appear (p=0.023) and what is the common cause of rare diseases worldwide (p<0.001) was significant. Overall scores were calculated, and only four (1.2%) participants answered all seven questions correctly that assessed knowledge of rare diseases (Table 3). Overall, the score of knowledge on rare diseases was not significant across healthcare professionals' occupations (p=0.182). Physicians had the highest overall average score of the population, and nurses had the lowest overall average score (Figure 2). Spearman's test was used to examine the correlation between self-declared knowledge and the total achieved score. There was a weak correlation (r=0.190; p<0.001), which indicated that the population's self-declared knowledge did not portray their actual knowledge on rare diseases. 

**Table 3:** Overall scores of participants across occupations.

Overall Score	Physicians (n=84)	Nurses (n=179)	Allied Health (n=70)	Total (n=333)
0/7 (0%)	15 (17.9%)	71 (39.7%)	22 (31.4%)	108 (32.4%)
1/7	30 (35.7%)	42 (23.5%)	20 (28.6%)	92 (27.6%)
2/7	22 (26.2%)	36 (20.1%)	17 (24.3%)	75 (22.5%)
3/7	7 (8.3%)	10 (5.6%)	3 (4.3%)	20 (6.0%)
4/7	5 (6.0%)	5 (2.8%)	2 (2.9%)	12 (3.6%)
5/7	3 (3.6%)	5 (2.8%)	3 (4.3%)	11 (3.3%)
6/7	1 (1.2%)	7 (4.0%)	3 (4.3%)	11 (3.3%)
7/7 (100%)	1 (1.2%)	3 (1.7%)	0	4 (1.2%)
Average	1.70±1.5	1.41±1.7	1.49±1.6	1.50±1.6

## Discussion

Evidence-based practice relies heavily on scientific literature, which presents advancements in the gold standards of care and may even present clinical guidelines for optimal patient care. Evidence-based practice has been recognized worldwide and implemented to enhance the quality of social and healthcare services and patient care [11]. To initiate evidence-based practice, information must come from instruments that assess the knowledge and awareness of healthcare professionals, thus providing the most convenient action plans to improve patient care. In this study, the instrument developed by Walkowiak D and Domaradzki J [7-9] was used to assess the knowledge and awareness of healthcare professionals about rare diseases. Core competencies were measured using self-declaration of knowledge and through performance or quiz-based questions to compare self-declared knowledge and actual knowledge [12]. Literature shows that medical practice encourages healthcare professionals to have certainty, making their self-declaration be presented higher, especially regarding patient care [13]. In some studies, it has been indicated that doctors ignore their uncertainty and prefer to present certainty in the presence of their patients. Katz J [14] argues that doctors habitually suppress their uncertainty to maintain patient confidence, Quill TE and Suchman AL [15] referred to an 'illusion of certainty' in medicine, and Atkinson P suggests that doctors are trained for certainty and that their medical training makes uncertainty difficult to acknowledge [16]. In our study, more than half of the participants across all occupations declared that they had moderate knowledge about rare diseases (Figure 1). However, the results of their performance on the quiz showed that most of the population had poor knowledge about rare diseases (Table 3). The relationship between self-declared knowledge and actual knowledge was statistically significant (p=0.005). The results seen throughout the questionnaire portray that the respondents lack knowledge since the highest rate of responses for most questions was "I don't know" (Table 2). However, it was shown that over half of respondents were aware that the common cause of rare diseases is genetic (p<0.001). Additionally, respondents were knowledgeable that rare diseases are most likely to appear in children (p=0.023). These results align with the literature, which states that, generally, the knowledge of rare diseases is poor, with correct answer rates for various questions varying from 2% to 91% [17]. In our study, the rates of correct answers varied from 8.7% to 53.5%. Similar to most studies, the highest rate of correct answers was seen in the question about the common cause of rare diseases [17]. Furthermore, the majority (92.8%) of respondents presented that they wanted to broaden their knowledge about rare diseases. As such, action plans to broaden the healthcare workers' knowledge of rare diseases will be developed. 

Initially, the short-term action plan is to present continuing medical education lectures to increase awareness about rare diseases and the availability of resources that healthcare professionals can use. A form of learning is using case presentations to teach about rare diseases, or problem-based learning. A study done on medical students found that engagement during learning achieves successful outcomes [18]. In addition, they found that engagement occurs when the case presented builds on previous knowledge, encourages interaction and thinking processes, offers challenging situations, and provides satisfaction from the learning experience. We believe that this may benefit our population, as the environment of King Hamad University Hospital and Bahrain Oncology Centre promotes continuing medical education lectures. Other routes to enhance knowledge about rare diseases could include attending academic conferences. In a study conducted across multiple hospitals, it was determined that the most common and effective methods of learning about rare diseases come from clinical work and academic conferences [19]. Furthermore, we aim to focus primarily on nurses as they play a pivotal role in assisting patients and their families within the healthcare system and also serve as nursing educators in our healthcare community. Our study revealed that nurses possess the lowest knowledge about rare diseases, with an overall average score of 1.41±1.70 compared to the population's overall average score of 1.50±1.60. As such, there is an urgent need to prepare practice guidelines for nurses, equipping them to make informed decisions about patients with rare diseases [8]. The long-term action plan aims to enhance the knowledge of rare diseases among healthcare professionals and the broader healthcare community through scientific research. Ultimately, the goal is to establish a national registry for all patients diagnosed with rare diseases. Registries serve as a source of real-world data, showcasing a range of clinical and biological features [20]. Regionally, the United Arab Emirates has initiated the development of a database encompassing genetic diseases found in the region [21]. As of April 2023, this database contains 2,299 entries of genetic diseases, 2,126 entries of genes, and 4,160 variants. The healthcare community, which includes not only healthcare professionals but the patients and the general public, also plays a role in increasing the awareness and knowledge of rare diseases. In a report published in 2020, the Kingdom of Bahrain was highlighted as a case study for rare diseases. The report stated that the biggest challenges with regard to rare diseases in Bahrain are that no rare disease registries are available at a national level, there are barriers to the creation of rare disease alliances, and there are no patient-based organizations that focus on rare diseases. Socio-cultural factors also pose a challenge to the progress of rare diseases. The report stated that there is a general lack of understanding of rare diseases and difficulties with integration due to social stigma and shame [22]. Action plans on a national level should include legislation to confirm the definition and classification of rare diseases, assembling epidemiological data on rare diseases, and international cooperation in research on rare diseases and the development of orphan drugs [23]. Scientific research is a vital tool to determine the needs of the rare diseases population. We aim to have a specific focus on scientific research in our action plan as it is needed to develop, build, and find solutions to the challenges identified in improving high-quality patient care [24].

In the future, we recommend that researchers test healthcare professionals' knowledge before and after an implemented intervention. This may increase researchers' understanding of what type of intervention style suits their work lifestyle, leading to a focus on how to increase their knowledge. We also recommend that researchers compare senior healthcare professionals and current healthcare students to present data on the current development of medical education. Lastly, we recommend that researchers provide a translated version of the questionnaire as per their population's knowledge to not only increase their sample size but also allow researchers worldwide to compare levels of knowledge on rare diseases. Eventually, this would help create a worldwide standard for education for rare diseases. Like other studies conducted, our study had several limitations. Firstly, healthcare professionals answered the questionnaire based on their memory, potentially leading to less accurate results [19]. Other limitations are related to the sampling methods for which convenience sampling was used, which may introduce bias and limit the generalizability of the findings. However, this was the most appropriate approach for this study based on the sample population. Additionally, the study had a limited sample size, as it was not conducted on a national scale. Furthermore, a significant challenge we faced was the lack of published literature on prevalence rates and the most common rare diseases specific to the Middle East and North African region.

## Conclusions

In conclusion, although our hospital's general knowledge about rare diseases may be lacking, it is vital to recognize that this is consistent with the global knowledge landscape. Therefore, it is crucial to prioritize rare disease education in both medical and nursing curricula as well as in continuing medical education programs. Moreover, strengthening advocacy efforts is essential to ensure the delivery of optimal patient care services in our region and beyond. By doing so, we can bridge the knowledge gap and improve healthcare outcomes for individuals affected by rare diseases. Integrating dedicated modules or courses on rare diseases within the core curriculum of medical and nursing programs, providing hands-on clinical exposure to patients with rare diseases through specialized clinics or partnerships with rare disease centers of excellence, inviting guest lecturers, including rare disease specialists, patient advocates, and researchers, to share their expertise and experiences with students, promoting research opportunities in rare diseases to foster a deeper understanding and encourage evidence-based practice, and creating awareness campaigns and events to engage the broader healthcare community can all emphasize the importance of rare disease education. These are specific ways that policymakers can adopt to underscore rare disease education.
